# OncomiR-17-5p: alarm signal in cancer?

**DOI:** 10.18632/oncotarget.19331

**Published:** 2017-07-18

**Authors:** Madhusudhan Reddy Bobbili, Robert M. Mader, Johannes Grillari, Hanna Dellago

**Affiliations:** ^1^ Department of Biotechnology, BOKU–University of Natural Resources and Life Sciences, Vienna, Austria; ^2^ Christian Doppler Laboratory on Biotechnology of Skin Aging, Department of Biotechnology, BOKU–University of Natural Resources and Life Sciences, Vienna, Austria; ^3^ Department of Medicine I, Comprehensive Cancer Center of the Medical University of Vienna, Vienna, Austria; ^4^ Evercyte GmbH, Vienna, Austria; ^5^ TAmiRNA GmbH, Vienna, Austria

**Keywords:** miRNA, miR-17-5p, biomarker, cancer

## Abstract

Soon after microRNAs entered the stage as novel regulators of gene expression, they were found to regulate -and to be regulated by- the development, progression and aggressiveness of virtually all human types of cancer. Therefore, miRNAs in general harbor a huge potential as diagnostic and prognostic markers as well as potential therapeutic targets in cancer.

The miR-17-92 cluster was found to be overexpressed in many human cancers and to promote unrestrained cell growth, and has therefore been termed onco-miR-1. In addition, its expression is often dysregulated in many other diseases. MiR-17-5p, its most prominent member, is an essential regulator of fundamental cellular processes like proliferation, autophagy and apoptosis, and its deficiency is neonatally lethal in the mouse. Many cancer types are associated with elevated miR-17-5p expression, and the degree of overexpression might correlate with cancer aggressiveness and responsiveness to chemotherapeutics – suggesting miR-17-5p to be an alarm signal. Liver, gastric or colorectal cancers are examples where miR-17-5p has been observed exclusively as an oncogene, while, in other cancer types, like breast, prostate and lung cancer, the role of miR-17-5p is not as clear-cut, and it might also act as tumor-suppressor.

However, in all cancer types studied so far, miR-17-5p has been found at elevated levels in the circulation. In this review, we therefore recapitulate the current state of knowledge about miR-17-5p in the context of cancer, and suggest that elevated miR-17-5p levels in the plasma might be a sensitive and early alarm signal for cancer (‘alarmiR’), albeit not a specific alarm for a specific type of tumor.

## INTRODUCTION

The role of miRNAs in human development, homeostasis and disease is by now well acknowledged. Especially in the context of cancer, a large set of studies has by now accumulated which shows the role of some miRNAs as bona fide oncomiRs. Among these, the miRNA-17-92 cluster seems of special interest as it has been the first oncomiR to be described, but one of the cluster members, miR-17-5p, has also been found to decrease with aging and might even prolong the life span of mice upon overexpression. With this in mind, we set out to summarize the current knowledge of miR-17-5p in the context of cancer. We thereby surprisingly found that it is elevated in the serum or plasma of a large variety of solid and hematologic tumor types, which prompts us to here postulate a function of circulating miR-17-5p as an alarm signal that is sensitive for tumors in general, albeit not specific for a defined tumor type. Such a biomarker, however, might be useful to prompt physicians to demand a thorough clinical check-up of individuals for early cancer detection.

### Biogenesis and function of miRNAs

**Figure 1:**
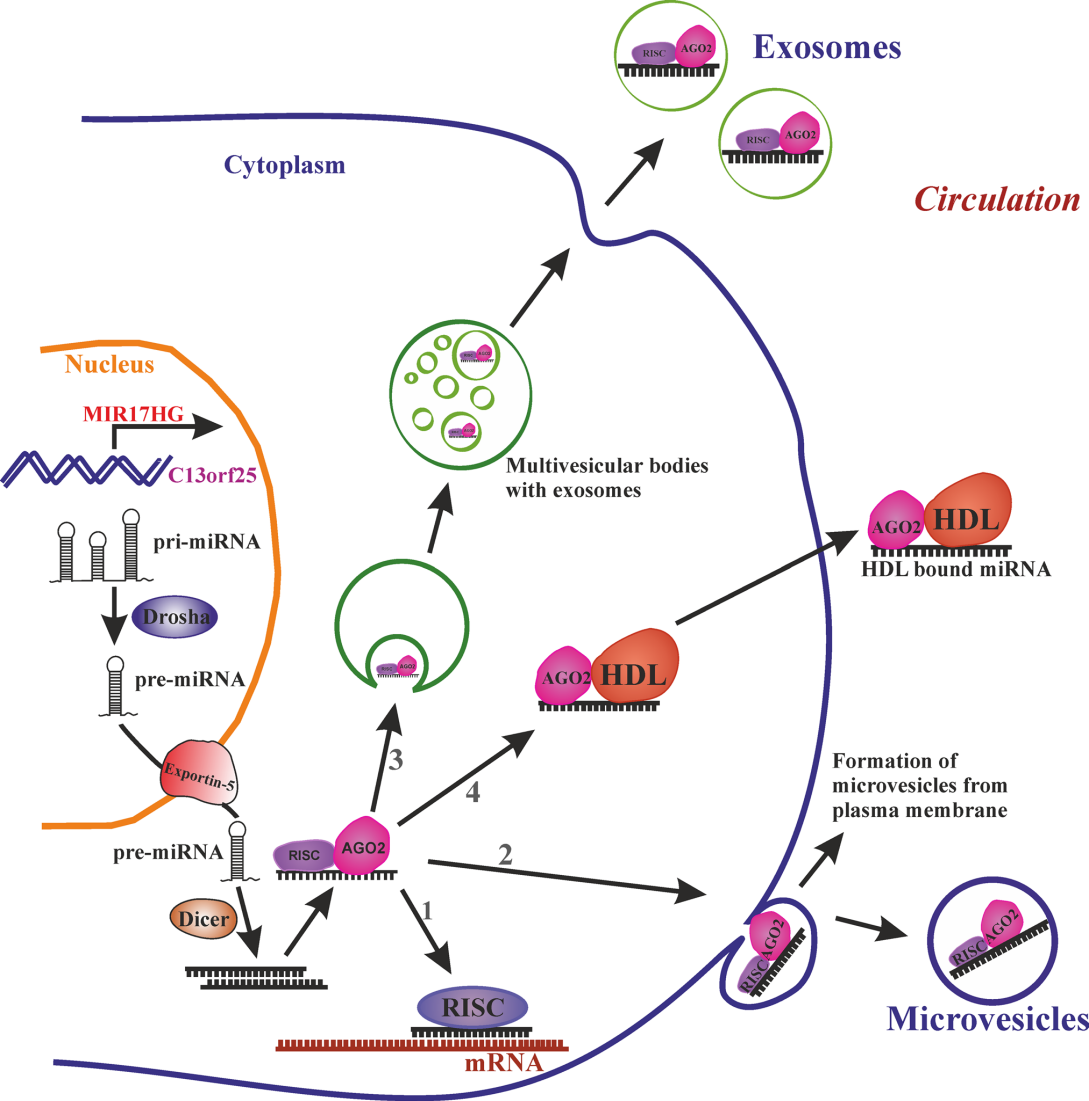
miRNA biogenesis and release into circulation Transcribed primary miRNA (Pri-miRNA) processed to precursor miRNA (Pre-miRNA) in the nucleus is exported to cytoplasm by exportin-5. In the cytoplasm pre-miRNA is processed to mature miRNA by Dicer. The mature miRNA further (1) can target the mRNA in cytoplasm, or bind to RNA-binding protein Ago-2 and release to circulation via (2) loading the miRNA-Ago2 complex to microvesicles which are formed by budding of plasma membrane or (3) loaded in to smaller vesicles called exosomes which are formed by endosomal invagination, can form multivesicular bodies (MVB) and upon fusion of MVBs to plasma membrane exosomes are released into circulation or (4) miRNA-Ago2 complex can directly interact with high-density lipoproteins (HDL) and be released into circulation.

MiRNAs are a class of small non-coding silencing RNAs of approximately 22 nucleotides in length which have a significant role in regulating gene expression. miRNAs bind to complementary regions in the mRNAs of protein-coding genes and mediate translational silencing or decay of their targets. miRNAs are encoded by intergenic regions or by intronic or even exonic regions of other genes and transcribed as a long primary miRNA (pri-miRNA) and processed to precursor miRNA (pre-miRNA) in the nucleus by Drosha [1]. Then they are exported to the cytoplasm by Ran-GTP and Exportin-5 where they are processed to mature microRNA (miRNA) by the type III RNAse Dicer [2, 3] (Figure 1). Members of a specific cluster can also be processed in a context-dependent manner, as explained by Cáceres JF *et al.*, where miR-18a stability is changed by hnRNP A1 (Heterogeneous Nuclear Ribonucleoprotein A1) in comparison to the other cluster members [4]. The first miRNA discovered was lin-4 in *Caenorhabditis elegans* [5] and was at first considered a nematode peculiarity. Only after discovery of let-7 and determination of its evolutionary conservation [6] was the door opened for the discovery of a whole new world of non-coding RNAs (ncRNAs) well beyond tRNAs, snRNAs or snoRNAs, comprising so far more than 2500 known mature miRNAs produced from nearly 2000 individual miRNA precursors in the human genome. Gradually, miRNAs turned out to form an entirely new layer of complexity that modulates and regulates virtually all aspects of cellular and organismal life.

miRNAs regulate gene expression of target genes post-transcriptionally by a ‘loose specificity binding’ manner. This binding depends on the “seed” region consisting of nucleotides 2–8 of the miRNA, and additional interactions with other regions of the miRNA stabilizes this interaction [7]. Thus, one miRNA is able to regulate up to 100 mRNA targets and therefore potentially orchestrates a large variety of cellular processes similar to transcription factors [8–10] and post-transcriptional operons [11]. There are two proposed models of how miRNAs target mRNAs, the standard model and the expanded model [12–15].

According to the “standard” model, miRNA and target mRNA form exact, that is, Watson–Crick base pairs absent of any bulges and wobbles in the seed region. The “expanded” model additionally allows wobble base pairing between U and G and creation of bulges either on the miRNA or the target mRNA side. Members of miR-17-92 cluster have at least two G/U bases in their seed region and therefore potentially bind to their targets according to the expanded model.

One of the best-studied set of miRNAs so far are the miR17-92 cluster members. This cluster contains 6 miRNAs with each of them having specific roles. Here in this review we focus on one of its member, miRNA-17-5p, and present current state of knowledge in the context of cancer, plasma or serum levels for specific type of tumors making it an ‘alarm signal’ for early detection of tumors.

### Circulating miRNAs

Over the past decade, circulating miRNAs have emerged as promising biomarkers for a broad spectrum of age-associated diseases. In one cross-sectional study, circulating miRNA profiles were able to discriminate osteoporotic fracture patients from non-fractured individuals [16]. In the circulation miRNAs are rescued from RNase degradation either by extracellular vesicles (EVs), by RNA-binding proteins or by associating with apolipoproteins. EVs like exosomes (30–100 nm), or microvesicles (100–1000 nm) play an important role in cell-to-cell communication by carrying miRNA, proteins, metabolites etc., from the cell of origin to a target cell. EVs can be loaded with miRNA and released into circulation by mechanisms like the ceramide-dependent secretory machinery, the tetraspanin or ESCRT (endosomal sorting complexes required for transport) transport machineries. However, not all the circulatory miRNAs are loaded into EVs, as a large number of miRNAs in the circulation are associated with Ago2 (Argonaute 2) protein, one of the subunit of the RNA-induced silencing complex [17]. Alternatively, miRNAs can be associated with HDL (High-density Lipoprotein) molecules which not only transport, but also target miRNAs to their recipient [18] (Figure 1).

### Transcriptional regulation and target mRNAs of miRNA-17-92 cluster and miR-17-5p

#### Transcriptional regulation of the miRNA-17-92 cluster

The locus of the miR-17-92 cluster is on chromosome 13 in the non-protein-coding gene MIR17HG (the miR-17-92 cluster host gene) within the open reading frame 25 (C13orf25). The miR-17-92 cluster transcript comprises six miRNAs - miR-17-5p, miR-18a, miR-19a, miR-20a, miR-19b-1 and miR-92a-1 - and is highly conserved among vertebrates [19, 20]. Expression of miR-17 as well as its seed region is strongly conserved in higher animals. In humans paralogous versions are present in the miR-106a-363 and miR-106b-25 clusters which have supposedly been formed by intra-genomic gene duplication as reviewed previously [21].

Several transcription factors are involved in miRNA-17-92 cluster transcriptional activation. One well known transcriptional factor which regulates miR-17-92 cluster is the transcription factor c-Myc, an important proto-oncogene. C-myc is known to regulate 10–15% of genes in the human genome which are involved in a wide variety of functions like cell cycle, apoptosis, energy metabolism and macromolecular synthesis. In human cancer, c-myc mutations are most frequent [22, 23, 24]. C-myc not only activates the miR-17-92 cluster but simultaneously also prevents the abundance of mRNA by a negative feedback loop targeting genes which are also known or predicted targets of the miR-17-92 cluster like RPS6KA5 (ribosomal protein S6 kinase, 90 kDa, polypeptide 5), BCL11B (B-cell CLL/lymphoma 11B), PTEN and HCFC2 (host cell factor C2) [25, 26].

In addition, the E2F family of transcription factors like E2F1, E2F2 and E2F3 activates the genes that are involved in cell progression from G_1_ to S phase and are reported to be direct targets of miR-17-92 cluster. In parallel, there is a tight regulatory loop where E2F1 and E2F3 in specific can induce the transcription of the miR-17-92 cluster. Aurora kinase A (AURKA), a serine/threonine kinase is overexpressed in many cancer types. AURKA is a known upstream regulator of E2F1 by inhibiting it’s proteosomal degradation, thus promoting expression of the mir-17-92 cluster [27]. miR-17-5p and miR-20a in turn negatively regulate E2F1 expression [28].

In contrast, p53 acts as negative regulator of miR-17-92 cluster transcription. Under hypoxic conditions p53 represses transcription of the miR-17-92 cluster promoting hypoxia induced apoptosis [29]. In addition, the ENCODE (Encyclopedia of DNA Elements) project revealed additional transcriptional factors like BCL3 (B-cell CLL/lymphoma 3), IRF1 (Interferon Regulatory Factor 1), SP1 (Sp1 transcription factor), TAL1 (T-cell acute lymphocytic leukemia 1) and ZBTB33 (zinc finger and BTB domain containing 33) regulating the miR-17-92 cluster [30] (also reviewed by Mogilyansky & Rigoutsos [31] and Dellago *et al.* [21]).

In terms of ubiquitous transcription of miR-17-5p, it was found to be expressed in all 40 different normal human tissues tested including brain, muscle, circulatory, respiratory, lymphoid, gastrointestinal, urinary, reproductive and endocrine systems [32]. High level of expression was observed in thymus and lowest in PBMCs (peripheral blood mononuclear cells). Expression of miR-17-3p is approximately half of the level of miR-17-5p except for PBMCs, where expression was below detection limits [32]. MicroRNA expression and sequence analysis database (mESAdb) [33], which integrates data from several databases like e.g. the one by Basekerville and Bartel [34] substantiates these findings and emphasize the importance of miR-17-5p in all tissues.

### Targets of miR-17-92 cluster and miR-17-5p

Experimentally confirmed targets of the miR-17-92 cluster are PTEN and E2Fs in the context of cell cycle progression and apoptosis [35]. Various studies report a wide range of targets of the miR-17-92 cluster like members of the TGFß (transforming growth factor-β) signaling pathway [36], BCL2L11 (BCL2 Like 11), IRF1, JAK2 (Janus Kinase 2), PKD1 (Polycystin 1, Transient Receptor Potential Channel Interacting), PKD2 (Polycystin 2, Transient Receptor Potential Cation Channel), RBL1 (RB Transcriptional Corepressor Like 1), and STAT3 [37–40]. Heinrich Kovar *et al.* elucidated the targets of miR-17-92 cluster in Ewing sarcoma and found CTGF (Connective Tissue Growth Factor), FOSL2 (FOS Like 2, AP-1 Transcription Factor Subunit), GBP3 (Guanylate Binding Protein 3) and SERPINE1 (Serpin Family E Member 1) are effectively targeted by cluster [41]. It was reported by Felsher *et al.* that miR-17-92 cluster can target specific chromatin regulatory genes, such as Sin3b (SIN3 transcription regulator family member B; a transcriptional repressor for MYC-responsive genes), Hbp1, Suv420h1 (suppressor of variegation 4–20 homolog 1; a histone methyltransferase, targeted to histone H3 by retinoblastoma proteins), and Btg1 (B-cell translocation gene 1, anti-proliferative; a regulator of cell growth and differentiation) [42], as well as the apoptosis regulator Bim (Bcl-2 interacting mediator of cell death; an activator of neuronal and lymphocyte apoptosis) [42–44]. miR-17-92 cluster seems to target the genes involved in maintenance of cell proliferation and survival. We summarize confirmed targets of miR-17-5p by luciferase reporter assay in Table 1.

**Table 1:** Validated gene targets of miR-17 and pathways affected by their regulation in cancers

Pathology	Process	Pathways affected	Targets of miR-17	References
Aging	Autophagy	MKP7/mTOR pathway	ADCY5	[47]
Organ aging	Autophagy	FoxO3a and LC3B pathways	IRS-1	[47]
	Heart failure	Matrix remodelling	TIMP1, TIMP2	[126]
	Cardiac aging	Par4/CEBPB/FAK signalling	Par-4	[127]
Prostate cancer	Tumor suppressor	antioxidant pathway in mitochondria	MnSOD, Gpx2, TrxR2	[118]
	Cell proliferation and invasion (Metastasis)	Matrix Metallopeptidase regulation	TIMP3	[117]
Hepatocellular carcinoma	Cell proliferation and migration (Metastasis)	PI3K pathway, glycosylation	PTEN, GalNT7, vimentin	[28, 63]
	Cell proliferation and migration (Metastasis)	p38-HSP27 signalling	E2F1	[63]
Breast cancer	Cell migration and invasion	Wnt/β-catenin pathway	HBP1	[67]
	Tumor suppressor (growth arrest)	IGF-1/AIB1 pathway	AIB1, E2F1	[68]
	Tumor suppressor	Translation initiation	PDCD4	[70]
	Tumor suppressor	Cell cycle	CCND1	[71]
	Tumor suppressor	PI3K pathway	PTEN	[70]
Lung cancer	Apoptosis	Initiation of autophagy	Beclin-1	[80]
Gastric cancer	Inhibition of apoptosis	cell proliferation	TP53INP1, P21	[86]
	Cell proliferation and migration	TGFß	TGFBR2	[88]
	Cell proliferation	Cytokine mediated signalling	SOCS6	[87]
Colorectal cancer	Cell proliferation and invasion (Metastasis)	GABBR1 signalling	GABBR1	[98]
	Cell cycle progression	Cytoskeletal organization	RND3	[97]
Osteosarcoma	Cell proliferation and differentiation	Wnt/β-catenin pathway	SMAD7	[105]
	Cell migration and invasion	Akt pathway	BRCC2	[106]
Leukaemia	Cell differentiation	Cytokine mediated signaling: JAK-STAT pathway	STAT3	[111]

Abbreviations: ADCY5, Adenylate Cyclase 5; AIB1, Amplified in breast cancer 1; BRCC2, Breast Cancer Cell Protein 2; CCND1, Cyclin D1; E2F1, E2F Transcription Factor 1; CEBPB, CCAAT/Enhancer Binding Protein Beta; FAK, Focal Adhesion Kinase; FOXO3a, Forkhead Box O3; GABBR1, Gamma-Aminobutyric Acid Type B Receptor Subunit 1; GalNT7, Polypeptide N-Acetylgalactosaminyltransferase 7; GPX2, Glutathione Peroxidase 2; HBP1, HMG-Box Transcription Factor 1; HSP27, Heat Shock 27kD Protein 1; IGF-1, Insulin Like Growth Factor 1; IRS1, Insulin Receptor Substrate 1; LC3B, Microtubule Associated Protein 1 Light Chain 3 Beta; MKP7, Mitogen-Activated Protein Kinase Phosphatase 7; MnSOD, Mitochondrial Superoxide Dismutase 2; mTOR, Mechanistic Target Of Rapamycin; PAR4, Prostate Apoptosis Response 4 Protein; PDCD4, Programmed Cell Death 4; PI3K, Phosphatidylinositol-4,5-Bisphosphate 3-Kinase; PTEN, Phosphatase And Tensin Homolog; RND3, Rho Family GTPase 3; SOCS6, Suppressor Of Cytokine Signaling 6; SMAD7, SMAD Family Member 7; STAT3, Signal Transducer And Activator Of Transcription 3; TGFBR2, Transforming growth factor-β receptor 2; TIMP1, TIMP Metallopeptidase Inhibitor 1; TIMP2, TIMP Metallopeptidase Inhibitor 2; TIMP3, TIMP Metallopeptidase Inhibitor 3; TP53INP1, Tumor Protein P53 Inducible Nuclear Protein 1; TXNRD2, Thioredoxin Reductase 2.

### miR-17-5p: a link between proliferation, cancer and aging

miR-17-5p plays a different role in cancer and aging. Aging is a well known risk factor for many types of cancer prognosis. Inhibition of mTOR (mammalian target of rapamycin) slows aging and postpones age-related diseases like diabetes, cancer and cardiovascular diseases and widely accepted aging model [45] by activating autophagy. Autophagy helps in clearance of unnecessary molecules or organelles and nutrient provision by degradation of intracellular pathogens where autophagic potential was lost in normal and premature aging [46]. During the process of aging, autophagy maintains cellular function by removing protein aggregates and allowing degradation of aged cellular components [45]. Two regulatory loops exist where mTOR is inhibited in autophagy. On the one hand, miR-17-5p inhibits mTOR by inducing MKP7 (Mitogen-Activated Protein Kinase Phosphatase 7) via targeting ADCY5 (Adenylate Cyclase 5): Upon dephosphorylation of mTOR by MKP7, mTOR dimerizes with PRAS40 (40-kDa proline-rich AKT substrate) and gets inhibited [21, 47]. On the other hand miR-17-5p targets IRS1 thus activating AMPK (AMP-activated protein kinase) which stops phosphorylation of ULK1 (Unc-51 like autophagy activating kinase 1) by mTOR and promotes formation of ULK1-ATG13-FIP200 (ATG13, autophagy related 13; FIP200, focal adhesion kinase family kinase-interacting protein of 200 kDa) complex required for the initiation of autophagy, a major complex involved in the formation of autophagosome [21].

In many types of cancer deregulation of mTOR is observed, which is a central regulator of cell proliferation. mTOR inhibitors like Rapamycin and its analogs are widely used as potential anti-tumour agents, some already approved for clinical use in cancer therapy. mTOR plays an important role in cell physiology and tissue maintenance, and use of its inhibitors like rapamycin leads to up-regulation of the miR-17-92 cluster and down-regulation of tumor suppressors [48]. Inhibitors of miR-17 could potentially serve as adjuvants in chemotherapy as oncogenic miRNAs like miR-17 are upregulated in rapamycin-resistant cells and inhibition of miR-17 restored rapamycin sensitivity. For details on miR-17-5p’s role in aging, please refer to a recent review [21].

### miR-17-5p and its role in cancer

Evidence from many different tumors support the idea that miR-17-5p is an oncogene, even though its other cluster member, miR-18a is considered the most oncogenic [49]. Large-scale miRnome analysis on 540 samples including lung, breast, stomach, prostate, colon and pancreatic tumors identified miR-17-5p as upregulated in all solid tumors [50]. Its overexpression in hamster derived tumor cells also increases proliferation and protein production [51]. Due to the oncogenic properties of the miR-17-92 cluster, its members were also considered to be oncogenic. By now a more differentiated view has emerged, as miR-17-5p alone, by stimulating T cells can suppress cancer growth [52], while still able to drive hepatocellular carcinoma in a transgenic mouse model. In addition, it seems to have metastasis suppressor functions as well, at least by suppressing epithelial-to-mesenchymal-transition (EMT) and increasing tissue adherence and thus potentially inhibiting metastatic spreading of basal-like breast tumor cells [53]. On the other hand overexpression of miR-17 promotes the cancer cell migration by reducing cell adhesion and promoting cell detachment in immortalized rat prostate endothelial cells [54]. It was found that patients suffering from several different types of cancer have high circulating miR-17-5p levels in serum [55, 56], implying that increased serum levels of miR-17-5p could be an alarm signal for different types of cancers. Hence oncomiR-17-5p might be termed ‘alarmiR’.

The effect of miR-17-5p is highly dependent on many factors like type of cancer, model systems used and constructs used in model systems for knockdown or overexpression, as well as on the relative expression levels of miR-17-3p and miR-17-5p which was discussed in few cancer types where miR-17-3p did have synergistic or rescue effect. While we here focus on the role of the single miR-17-5p in formation and progression of distinct cancer types, Xiang and Wu [57] have reviewed the tumor-suppressive and tumorigenic properties of the miR-17-92 cluster as a whole.

### Hepatocellular carcinoma

Emerging evidence indicates that the miR-17-92 cluster and specifically miR-17-5p play an important role in carcinogenesis in the liver.

A liver-specific miR-17-92 transgenic mouse showed significantly increased hepatocellular cancer development. These results were complemented by overexpression of the miR-17-92 cluster in cultured human hepatocellular cancer cells, which enhanced proliferation, colony formation and invasiveness *in vitro*, whereas inhibition of the miR-17-92 cluster had the opposite effect [58].

MiR-17 might be largely responsible for the effect of the cluster, since overexpression of pre-miR-17 in a transgenic mouse model results in hepatocellular carcinoma (HCC). In addition, both miR-17-5p and miR-17-3p are abundantly processed from precursor miR-17 and have synergetic effects on developing HCC by binding different targets on different signaling pathways: miR-17-5p targets PTEN, one of the most frequently lost tumor suppressor in human cancers, while miR-17-3p represses expression of vimentin, an intermediate filament with the ability to modulate metabolism, and GalNT7, an enzyme that regulates metabolism of liver toxin galactosamine. These three proteins work in separate signaling pathways, but independently contribute to regulating proliferation and migration [59]. Thereby, miR-17-5p also targets the long non-coding RNA PTENP1, a pseudogene of PTEN. When overexpressed, PTENP1 sequesters miR-17, which would otherwise target PTEN and the negative Akt-regulator PHLPP (PH Domain And Leucine Rich Repeat Protein Phosphatase). Hence PTENP1 functions as miR-17 antagonist, representing an appealing approach for HCC treatment based on miR-17 function in tumorigenesis [60].

MiRNA-17-5p expression is highly elevated in patient-derived HCC tissues, especially in metastasis derived tissues when compared to controls [61]. This correlates with the observation that serum levels of circulating miR-17-5p were upregulated in a relapse group of patients and downregulated in the post-operative group. In addition, serum levels of miR-17-5p were associated with metastasis status and staging, suggesting that the miRNA in the serum indeed is tumor cell derived [62].

**Figure 2:**
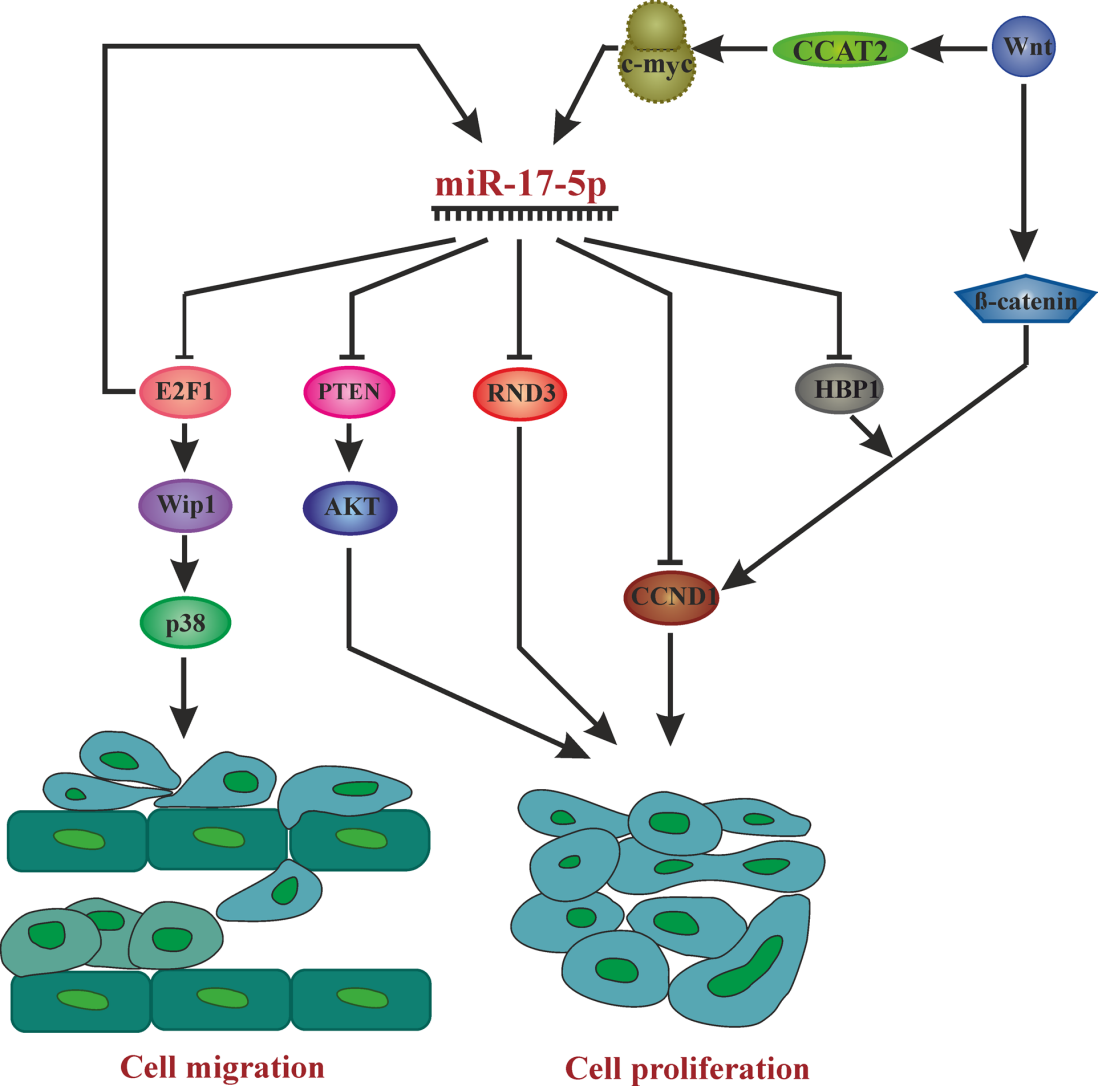
Overview of pathways affected by miR-17-5p in different cancer phenotypes leading to cell proliferation and migration AKT: Proto-oncogene c-Akt, c-myc: V-Myc Avian Myelocytomatosis Viral Oncogene Homolog, CCAT2: Colon Cancer Associated Transcript 2, E2F1: E2F transcription factor 1, CCND1: Cyclin D1, HBP1: HMG-Box Transcription Factor 1, P38: Mitogen-Activated Protein Kinase 14, PTEN: Phosphatase And Tensin Homolog, RND3: Rho Family GTPase 3, Wnt: wingless-type MMTV integration site family, Wip1: Protein Phosphatase, Mg2+/Mn2+ Dependent 1D.

HCC cell lines overexpressing miR-17-5p injected either subcutanously or into the livers of nude mice generating an orthotopic intrahepatic tumor model, miR-17-5p supported tumor growth and intrahepatic metastasis [63]. This was due to activating the p38 MAPK-HSP27 pathway by directly targeting the transcription factor E2F1, a transcriptional regulator of Wip1, which dephosphorylates and thus deactivates p38 (Figure 2). The p38 MAPK-HSP27 pathway mediates miR-17-5p’s effect on migration, but, however, is not involved in its effect on proliferation.

Summarized, miR-17-5p possesses oncogenic activity in the context of hepatocellular carcinoma.

### Breast cancer

Cumulative data clearly point to a role of miR-17-5p in the development and progression of breast cancer, and is currently being explored as biomarker for diagnosis, prognosis and therapeutic target.

qPCR-based miRNA expression profiling revealed that miR-17-5p, miR-18a-5p and miR-20a-5p exhibit enhanced expression in tissue samples derived from triple-negative as compared to luminal A breast tumors, which are less aggressive and have much better prognosis as well as lower recurrence rate [64]. In addition, Lehmann and co-workers studied miR-17 -among other miRNAs- as potential molecular marker to evaluate grade, receptor status and molecular type in breast cancer. Six miRNAs and five mRNAs were analyzed pairwise and examined for a possible correlation with histological breast cancer groups. The miR17/miR27b pair best discriminated samples with different tumour grades, but others correlated better with lymph node status, tumor size and oestrogen/progesterone receptor status, so that multiple marker pairs are required to characterize a tumor sample [65].

For a comprehensive review on the use of miRNAs as biomarkers for prognosis, diagnosis, therapeutic prediction and therapeutic tool in breast cancer, please refer to Bertoli *et al.* [66], who also discuss the potential of miR-17-5p as potential diagnostic biomarker.

Even though correlating miR-17-5p expression levels with various tumor properties might be very useful in the development of biomarkers, it does not give evidence about its tumorigenic or tumour-suppressive potential. After all, elevated miR-17-5p expression could either contribute to tumor formation and progression, or could represent a defense mechanism that is intended to limit carcinogenesis. So, far there exists evidence for both explanatory approaches.

According to Li *et al.* [67], miR-17-5p promotes human breast cancer cell migration and invasion through suppression of HMG box-containing protein 1 (HBP1), which they confirmed as a direct target of miR-17-5p. HBP1 is a component of the Wnt/β-catenin signaling pathway, which is frequently mutated in various cancer types (Figure 2). They found that miR-17-5p was highly expressed in strongly invasive, but not in weakly invasive BC cells, and that miR-17-5p overexpression enhanced migratory and invasive abilities of BC cells, while its downregulation had the opposite effect. Apart from promoting breast cancer cell migration and invasion by miR-17-5p, Liao XH *et al.* showed that miR-17-5p also promotes cell proliferation by down-regulating p21 which is a direct target of miR-17-5p in ERα (Estrogen receptor α) -positive breast cancer cells. ERα plays an important role in cell-cycle progression by promoting the expression of PCNA and Ki-67 along with miR-17-5p. Downregulation of p21 by miR-17-5p in turn promotes PCNA (proliferating cell nuclear antigen) activity, where p21 is a negative regulator of PCNA and thus ERα promotes breast cancer cell cycle progression and proliferation in p21/PCNA/E2F1-dependent pathway [68].

In contrast, miR-17-5p was described as tumor suppressor [69]. Downregulation of AIB1 (“Amplified in breast cancer 1”) by miR-17-5p decreased proliferation and abrogated insulin-like growth factor 1-mediated, anchorage-independent growth of breast cancer cells. A recent study from Liao XH *et al.* also established that miR-17-5p acts as a tumor suppressor by directly targeting STAT3 and inducing apoptosis in breast cancer cells by inhibiting STAT3/p53 pathway [70]. This shows how miR-17-5p tightly regulates the genes involved in cell proliferation and cell apoptosis.

Similarly, miR17-5p was identified as metastatic suppressor of basal-like breast cancer [53]. Out of 4000 genes linked to BC progression, miR-17-5p was confirmed *in vitro* and *in vivo* as regulator of multiple pro-metastatic genes, hence had an anti-metastatic effect, while miR-17-5p inhibition in BC cells enhanced expression of pro-metastatic genes and accelerated lung metastasis from orthotopic xenografts. Therefore, the authors suggest miR-17-5p as a potential therapeutic target for treatment of basal-like breast cancer.

The therapeutic potential of miR-17-5p inhibition in triple negative BC (TNBC), one of the most aggressive breast cancer forms, was also assessed as a therapeutic target [71]. Assuming that miR-17-5p inhibition would restore protein expression of tumor suppressive miR-17-5p targets Programmed cell death 4 (PDCD4) and Phosphatase and tensin homolog (PTEN), human TNBC cells were transfected with antisense oligonucleotides against miR-17-5p. The results showed that miR-17-3p seems to act as a back-up mechanism of miR-17-5p for these targets, and therefore, due to the high sequence homology between the antisense molecules and miR-17-3p, as well as to excess binding sites for miR-17-3p on the 3′UTR of PDCD4 and PTEN mRNAs, the antisense oligo acted as a miR-17-3p mimic and reduced PDCD4 and PTEN expression instead of restoring it.

In support of miR-17-5p’s tumor-suppressive role, recent bioinformatics and *in vitro* analysis revealed that levels of miR-17-5p are decreased in triple negative breast cancer cells resulting increase in CCND1 (cyclin D1) levels which is reason for uncontrolled proliferation. Expression of CCND1 was inhibited by overexpression of miR-17-5p [72]. Circulatory/serum miR-17-5p levels are deregulated which also reflects the differential biology of breast cancer subtypes [73]. Hence it even acts as a biomarker even to predict the stage of cancer.

Summarized, the tumorigenic or tumor-suppressive functions of miR-17-5p might depend on the cellular context, that is, on the model system used, cell type, cancer stage and many other factors, like for example “BRCA-ness”. De Summa *et al.* [74] show that overexpression of miR-17 in both mesenchymal-like BRCA1-proficient and in BRCA1- and BRCA2-mutated BC cell lines in addition to the significant overexpression of miR-17 in sporadic patients seems to suggest that downregulation of BRCA1, a presumed target of miR-17-5p mimics a ‘BRCAness’ phenotype, that is, a phenotype that some sporadic cancers share with BRCA1- or BRCA2-mutation carriers. Hence, miR17 might represent a biomarkers of ‘BRCAness’ phenotype, indicating which patients who could most benefit from PARP inhibitor therapies.

### Lung cancer

Several studies have investigated the relationship between miR-17-5p and lung cancer, mainly in view to its potential clinical application of miRNA expression profiles as diagnostic and prognostic marker.

For example, elevated miR-17-5p expression levels are present in tumor tissue and serum of lung cancer patients–including adenocarcinoma, squamous cell and adenosquamous carcinoma- compared to healthy controls. In addition, serum miR-17-5p levels were inversely related to the survival of patients with lung cancer, that is, high levels correlated with shorter survival times [75].

This is in contrast to studies that found miR-17 (no distinction between 5p and 3p) downregulated in lung adenocarcinoma initiating cells [76] and in non-small cell lung cancer (NSCLC). It should be mentioned though, that although miR-17-5p expression levels allowed distinction between NSCLC and healthy control, it was not useful as diagnostic marker for discriminating between NSCLC and chronic obstructive pulmonary disease (COPD) [77].

Other studies concluded that miR-17-5p expression levels did not have sufficient informative values to serve as diagnostic tool, at least using sputum miRNA profiling [78], This study confirms previous results of the same group [79], where miR-17-5p was not found either over-or under-expressed in human lung cancer.

In addition to exploring its diagnostic potential, miR-17-5p might also serve as therapeutic target in lung cancer treatment. According to Matsubara *et al.* [80], inhibition of miR-17-5p and miR-20a with antisense oligonucleotides (ONs) can induce apoptosis selectively in lung cancer cells overexpressing miR-17-92, suggesting the possibility of targeting an ‘oncomiR addiction’ to expression of these miRNAs in a subset of lung cancers. In marked contrast, antisense oligonucleotides against miR-18a, miR-19a or miR-92-1 led to no or slight inhibition of cell growth, indicating that single miRNAs of the miR-17-92 cluster have distinct roles on cancer formation and progression.

On the other hand, downregulation of miR-17-5p upregulates its target, the autophagy regulator beclin-1, which leads to apoptosis resistance of cancer cells upon paclitaxel treatment [81]. This is in accordance with the notion that miR-17-5p overexpression reduces cyto-protective autophagy by targeting Beclin-1 in paclitaxel resistant lung cancer cells [82]. To justify miR-17-5p acts as tumor suppressor, a study shows that low expression levels of miR-17 results in cisplatin resistance of NSCLC by high expression of CDKN1A (cyclin-dependent kinase inhibitor 1A) and RAD21 (Rad21 homolog (Schizosaccharomyces pombe)) [83]. Hence miR-17-5p plays a tumor suppressor role in this setting.

Thus, miR-17-5p can either promote or curb apoptosis of lung cancer cells. Again, the final effect of miR-17-5p seems to be highly context-dependent.

### Gastric cancer

Circulating miR-17-5p was found to be significantly elevated in the serum of patients with gastric cancer compared to healthy controls, and correlates with circulating tumor cells [84, 85]. However, a follow-up study failed to assign a prognostic value to miR-17-5p plasma levels, since there was a slight, but not significant difference in the survival rates of patient groups exhibiting low or high miR-17-5p plasma levels, although the trend might turn significant when based on larger sample size (*n* = 31 vs.38) [86]. This assumption has been verified by Wang *et al.* [55], they not only found that concentrations of miR-17-5p/20a were significantly associated with the differentiation status and tumor progression, but also revealed that high expression levels of miR-17-5p/20a were significantly correlated with poor overall survival. In addition, therapeutic potential for antagomirs against miR-17-5p/20a was suggested, which was applied as chemotherapeutics in a mouse tumor model. Indeed, levels of serum miR-17-5p/20a were notably reduced in post-treated mice with tumor volume regression.

A follow-up study from the same group investigated the cellular mechanisms involving miR-17-5p in gastric cancer and found that miR-17-5p/20a promote gastric cancer by directly targeting the tumor suppressors p21 and p53-induced nuclear protein 1 (TP53INP1), which results in unrestrained proliferation and apoptosis inhibition, respectively, and involve a positive regulatory circuit between miR-17-5p/20a and MDM2 (murine double minute 2). Their findings in gastric cancer cells were backed-up by administering antagomiRs against miR-17-5p/20a to reduce tumor formation in a xenograft mouse model [87].

Likewise, miR-17-5p increased the proliferation and growth of gastric cancer cells *in vitro* and *in vivo*, by targeting SOCS6, a cytokine-induced STAT inhibitor [88]. Another study shows that high levels of miR-17-5p decreased expression of its direct target TGFBR2 (transforming growth factor-β receptor 2), further promoting gastric cancer cell proliferation and migration [89]. Supporting the above studies, a clinical study states that serum levels of miR-17 from patients with gastric cancer are high compared to healthy individuals [90].

As mentioned in section 3.1, AURKA activates transcription of miR-17-92 by stabilizing the transcription factor E2F1. AURKA inhibitors are currently applied in clinical trials for treatment of gastrointestinal cancer [27], and since miR-17-92 represents one branch of AURKA-dependent oncogenic signaling, also direct inhibitors of miR-17-92 members might serve as potential targets in gastric and other types of cancer, but before that, more research on specific functions of single miR-17-92 members is required.

In summary, in the context of gastric cancer, miR-17-5p clearly acts as oncogene and targets the components of many pathways involved in cell proliferation and migration.

### Colorectal cancer

Among all the miRNAs of the miR-17-92 cluster, miR-17-5p showed highest expression in epithelial colon cells and expression levels increased in the transitional zone from normal to adenoma to adenocarcinoma (N-A-AC), suggesting a role in sequential evolution of early colon cancer [91].

Several studies confirm miR-17-5p overexpression in CRC (colorectal cancer) tissue samples [92, 93, 94]. Elevated miR-17-5p expression is also observed in early embryonic colon epithelium, and is sustained only in the proliferative crypt progenitor compartment. Downregulation of E2F1 by miR-17-5p is of importance for proliferation both during embryonic colon development and colon carcinogenesis [95].

What causes miR-17-5p overexpression leading up to CRC pathogenesis and by what targets does it regulate proliferation? The long noncoding RNA CCAT2, a WNT downstream target, induces miR-17-5p and MYC through TCF7L2 (Transcription Factor 7 Like 2) -mediated transcriptional regulation (Figure 2) [96]. Accordingly, miR-17-5p targets P130 (Retinoblastoma-Like 2, a presumed tumor suppressor, present in a complex that represses cell cycle-dependent genes) and subsequently activates the WNT/β-catenin pathway [97]. Hence, there exists a positive WNT signaling feedback loop involving miR-17-5p.

In addition, miR-17-5p directly targets RND3, a Rho Family GTPase that acts as tumor suppressor by promoting adhesion [98]. MiR-17 along with miR-106a/b and miR-20a/b targets GABBR1(gamma-amino-butyric acid type B receptor 1) thus promoting colorectal cancer cell proliferation and invasion [99].

What prognostic and therapeutic implications can be derived from miR-17-5p expression data? miR-17-5p expression levels might be used as predictive factor for chemotherapy response and a prognostic factor for overall survival in CRC, since patients with high miR-17-5p expression in tumor tissue have shorter overall survival rates [97, 100] and respond better to adjuvant chemotherapy than patients with low miRNA expression [97]. On the other hand, chemotherapy was found to further increase the expression levels of miR-17-5p in CRC cells *in vitro*, thereby repressing the pro-apoptotic factor PTEN and promoting chemoresistance [101]. A very similar observation was made in another tumor entity, pancreatic cancer, where an overexpressed nerve growth factor receptor (GFRα2) led to PTEN inactivation mediated by induction of miR-17-5p [102]. Downregulation of miR-17-5p by curcumin and its synthetic analogs inhibits CRC cell proliferation and induces apoptosis, and could provide the basis for future therapeutic approaches [103]. Supporting *in vitro* and tissue level high expression of miR-17-5p, a clinical study proves serum levels of miR-17 along with miR-19a, miR-20a and miR-223 were significantly upregulated in CRC patients compared to controls [104].

Briefly, miR17-5p plays a key role in colorectal cancer pathogenesis and progression. Henceforth miR-17-5p could be used as a diagnostic biomarker for colorectal cancer.

### Osteosarcoma

Expression of miR-17-5p is also high in osteosarcoma, whereby PTEN seems to be an important target contributing to progression and metastasis [105]. This seems in keeping with its role in osteoblastogenesis [106]. In addition to PTEN, SMAD7 and thus Wnt signalling is a direct target for miR-17-5p in this context. By targeting SMAD7, miR-17-5p promotes nuclear translocation of β-catenin, enhances expression of COL1A1 (Collagen Type I Alpha 1 Chain) and finally facilitates the proliferation and differentiation of femoral head mesenchymal stem (HMS) cells promoting osteonecrosis [106].

A very recent article explores the effects of miR-17-5p in osteosarcoma tumorigenesis and development. MiR-17-5p expression levels were associated with clinical stage, positive distant metastasis and poor response to neo-adjuvant chemotherapy. The tumor suppressor BRCC2, which is thought to induce apoptosis in a caspase-dependent manner, is a direct target of miR-17-5p [107]. Hence, miR-17-5p may be used as diagnostic and prognostic marker, but also as a potential target for molecular therapy of osteosarcoma.

Thus, in the context of the bone, miR-17-5p seems to have tumorigenic activity.

### Leukemia

Not only in solid tumors, but also in tumors of hematopoietic origin miR-17-5p is upregulated, like in both acute myeloid leukemia (AML) and chronic myeloid leukemia (CML). Expression profiling of acute myeloid leukemia (AML) identified a set of seven miRNAs comprising miR-17-5p that allows discrimination of three common AML-causing chromosomal translocations with a diagnostic accuracy of > 94%, and is significantly overexpressed in MLL (mixed lineage leukemia) rearrangements, which causes particularly aggressive leukemia with poor prognosis [108].

A study in multiple myeloma (MM) patients showed that high levels of miR-17-5p, miR-20a and miR-92-1 of miR-17-92 cluster are associated with shorter progression-free survival, suggesting poor prognosis [109]. Most interestingly, upon resistance to therapy of multiple myeloma with bortezomib, the exosomal transfer of several microRNAs seems to be altered, among them miR-17-5p, which was significantly reduced [110]. High levels of miR-17-5p, which further downregulate CDKN1A (Cyclin Dependent Kinase Inhibitor 1A), p21 and E2F1 tumor suppressor genes in imatinib sensitive and resistant chronic myeloid leukemia (CML) cells compared to peripheral blood mononuclear cells (PBMCs), have also been observed [111]. Hypoxia was suggested to induce differentiation of AML cells by mechanisms independent of transcription. Indeed, this was shown to happen via inhibition of miR-17-5p. HIF-1α (Hypoxia Inducible Factor 1 Alpha Subunit) downregulates the expressions of miR-17-5p and miR-20a through a mechanism that is dependent of c-Myc but independent of its transcription partner HIF-1ß*.* As p21 and STAT 3 are direct targets of miR-17-5p and miR-20a, downregulation of miR-17-5p and miR-20a induces myeloid differentiation and growth arrest in AML cells *in vitro* and *in vivo* [112]. This further supports that upregulation of miR-17-5p is at least associated to myeloid leukemia.

In lymphocytic leukemia, the available data is more ambiguous. According to Zanette *et al.* [113], the miR-17-92 cluster was upregulated in acute lymphocytic leukemia (ALL), but no cluster member was among the most highly expressed miRNAs in chronic lymphocytic leukemia (CLL). However, miR-17-5p was found downregulated in chronic lymphocytic leukemia both with normal p53 and with mutated/deleted p53, but downregulation was more pronounced in the latter patient group [114, 115]. Nonetheless, results derived from a SCID mouse model suggests the suitability of miR-17 as a therapeutic target for CLL treatment. This is due to results showing that antagomiR-17 strongly reduced tumor growth and increased survival when injected *in vivo* in tumors generated by MEC-1 cell injection into SCID mice [116]. How these contradictory findings could be reconciled is subject to further research. A comprehensive review discusses the roles of miRNAs in B-cell lymphoma with much emphasis on the miR-17-92 cluster [117].

### Prostate cancer

Conflicting results on tumor suppressor versus promoter function exist for prostate cancer (PC): Both mature miR-17-5p and passenger strand miR-17-3p target TIMP3 which has synergetic effect on enhancing prostate tumor growth and invasion [118]. However, high levels of miR-17-3p have also been reported to suppress tumorigenicity of PC cells through inhibition of mitochondrial antioxidant enzymes [119]. This effect seems mediated by p300/CBP-associated factor (PCAF) as a target of miR-17-5p modulating the androgen receptor transcriptional activity [120]. Androgen receptor (AR) signaling is critical for most aspects of prostate growth and tumorigenesis [120]. A potential anti-prostate cancer drug, glucosinolate-derived phenethyl isothiocyanate (PEITC), results in miR-17-5p-mediated suppression of PCAF and again AR-regulated transcriptional activity and cell growth of prostate cancer cells, suggesting a new mechanism by which PEITC modulates prostate cancer cell growth [121].

Resveratrol and Pterostilbene decrease the levels of endogenous as well as exogenously expressed miR-17, miR-20a and miR-106b thereby upregulating their target PTEN [122] and eventually leading to reduced tumor growth *in vivo*. According to a recent report, circulating exosomes from prostate cancer cells carry long non-coding RNAs which are themselves enriched with miRNA seed regions that can bind to let-7 and miR-17 families like a miRNA sponge [123]. This indicates that they are part of tumorigenic pathways and might find use as a therapeutic target and biomarker also in the context of prostate cancer.

In cancers like glioblastomas, under stress conditions miR-17 plays a dual role depending on the conditions. It acts as a tumor suppressor in normal growth conditions by inhibiting PTEN through miR-17-5p and at unfavorable conditions miR-17-3p promotes tumor cell survival by inhibiting MDM2 [124]. These results state that miR-17-3p also plays an important role in different cancers either in synergetic way or as rescue for miR-17-5p. Further studies on miR-17-3p are required to establish a firm regulation between miR-17-5p and miR-17-3p.

## CONCLUSIONS

The role of miR-17-5p as an oncomiR is supported by many studies, while also the opposite, a tumor suppressive role has been found in some studies. Therefore, its role seems to be cell type and tumor type dependent and more work in specific settings will be necessary to dissect all of its roles in oncology. In Figure 2, we summarize the pathways effected by miR-17-5p in different cancer types.

In the context of biomarkers, miRNAs are considered as promising emerging biomarkers in cancer, especially when considering circulating miRNAs as minimally invasive analytes within liquid biopsies [16]. We here have summarized studies that indicate that elevated levels of miR-17-5p might be an alarm signal for cancer, that might be sensitive, albeit not specific for a single type of cancer. Still, circulating miRNAs as biomarkers or alarmiRs still lack sufficient studies to be able to define the range of inter-individual variation in the general healthy population and consequently define thresholds for e.g. miR-17-5p in serum or plasma that would lead to the decision of careful follow up clinical testing for the presence of a tumor. Still, tissue based miRNA signatures have already reached the markets of diagnostics in cancer, e.g. Rosetta Genomics [125, 126] for determination of the primary tumor origin of metastasis, emphasizing that also circulating miRNAs might soon lead to biomarker signatures that can support clinical decisions.
